# Unraveling response to temozolomide in preclinical GL261 glioblastoma with MRI/MRSI using radiomics and signal source extraction

**DOI:** 10.1038/s41598-020-76686-y

**Published:** 2020-11-12

**Authors:** Luis Miguel Núñez, Enrique Romero, Margarida Julià-Sapé, María Jesús Ledesma-Carbayo, Andrés Santos, Carles Arús, Ana Paula Candiota, Alfredo Vellido

**Affiliations:** 1 Centro de Investigación Biomédica en Red, Bioingeniería, Biomateriales y Nanomedicina (CIBER-BBN), Cerdanyola del Vallès, Spain; 2 Intelligent Data Science and Artificial Intelligence (IDEAI-UPC) Research Center, Barcelona, Spain; 3 Department of Computer Science, Universitat Politècnica de Catalunya (UPC BarcelonaTech), Barcelona, Spain; 4 Universitat Autònoma de Barcelona, Cerdanyola del Vallès, Spain; 5 Institut de Biotecnologia i Biomedicina, Cerdanyola del Vallès, Spain; 6 Biomedical Image Technologies Laboratory (BIT), ETSI Telecomunicación, Universidad Politécnica de Madrid (UPM), Madrid, Spain

**Keywords:** Cancer, Computational biology and bioinformatics, Diseases, Oncology

## Abstract

Glioblastoma is the most frequent aggressive primary brain tumor amongst human adults. Its standard treatment involves chemotherapy, for which the drug temozolomide is a common choice. These are heterogeneous and variable tumors which might benefit from personalized, data-based therapy strategies, and for which there is room for improvement in therapy response follow-up, investigated with preclinical models. This study addresses a preclinical question that involves distinguishing between treated and control (untreated) mice bearing glioblastoma, using machine learning techniques, from magnetic resonance-based data in two modalities: MRI and MRSI. It aims to go beyond the comparison of methods for such discrimination to provide an analytical pipeline that could be used in subsequent human studies. This analytical pipeline is meant to be a usable and interpretable tool for the radiology expert in the hope that such interpretation helps revealing new insights about the problem itself. For that, we propose coupling source extraction-based and radiomics-based data transformations with feature selection. Special attention is paid to the generation of *radiologist-friendly* visual nosological representations of the analyzed tumors.

## Introduction

Glioblastoma (GB) is the most frequent of the aggressive primary brain tumor types found in human adults. Standard treatment for GB involves maximal resection surgery followed by radiotherapy and chemotherapy. For the latter, temozolomide (TMZ) is a common drug of choice^1^. Moreover, GB are heterogeneous tumors, showing high variability, although their prognostic is invariably bad: survival rates are in average 16–18 months after diagnosis. Such heterogeneity, in addition to the usual inter-patient variability, highlights the need of improvement of therapy response assessment, pursuing early and confident input information which may be useful for personalizing therapy schedules/strategies^2^. Therapy response follow-up is usually performed following strict guidelines, mostly centered in aspects such as tumor volume and contrast uptake using defined categorizations. Magnetic Resonance Imaging (MRI) is often used for the non-invasive evaluation of the GB response to therapy, through criteria such as the response assessment in neuro-oncology (RANO)^3^ and the Response Evaluation Criteria in Solid Tumors (RECIST)^4^, which are not exempt of misinterpretation due to pseudoresponse and pseudoprogression^5^. There is still much room for improvement in therapy response follow-up in GB, which can be at least partially addressed with preclinical GB models. Accordingly, this study addresses a preclinical question that involves distinguishing between treated and control (untreated) mice bearing GL261 GB tumors in a noninvasive manner using magnetic resonance-based data. Therapeutic protocols applied in this study have been proved to raise sustained transient or sustained response (and even cure) in GB-bearing mice^6^. In this sense, we consider that treated mice are responding to therapy.

It is worth noting that MR-based data come in different modalities: MRI is related to anatomical data (tumor volume, edema, contrast uptake), while Magnetic Resonance Spectroscopic Imaging (MRSI) is related to metabolomics data. In this work, MRI was not analyzed in the conventional RECIST approach. Instead, regions of interest (RoI) of the MRI were quantified using Radiomics, which entails the extraction of quantitative radiologic features in the form of image-based statistics that could be associated with clinical outcomes. If those features are sufficiently tumor type-specific, there is a chance to improve the predictive ability of images using their parsimonious summary description without resorting to the complete image^7^.

In addition to an exploratory, objective Radiomics study with MRI data, the current study amplifies the parametric scope to include MRSI data, since spectroscopy provides information about the molecular properties and metabolic heterogeneity of the tumor tissue^2^. This type of metabolomic information is not being taken into account in the clinical guidelines for therapy response follow-up. Previous research^6,8,9^ has shown that the metabolomic patterns extracted from MRSI can distinguish between treated and responding versus control murine GL261 GB. To this point, though, no attempt has been made to compare the capabilities of radiomics-based MRI data transformations and source extraction-based MRSI data transformations and its potential impact in future clinical studies. This study aims to do so, but it also aims to go beyond the comparison of methods for the discrimination of treated/responding and control murine GL261 GB tumors and provide an analytical pipeline that could be used in subsequent human studies.

Medical decision making is a well-honed combination of human expertise and available medical evidence. Due to technological and scientific advances, medicine is swiftly becoming a data-centered discipline. This datafication process becomes a unique opportunity for quantitative data analysis based on statistics and machine learning (ML). Even if so, it has been argued that ML and similar tools should only be used as an expert technological companion to the human expert decision making process. This is because, beyond providing enhanced accuracy, medical decision support systems must comply with existing guidelines, medical ethics and current legislation^10^. It has been claimed that for ML and similar methods to be accepted, legally compliant and put to work in the medical domain, they must be interpretable for the medical expert, so that the expert can explain, at least to some extent, the reasoning behind the algorithm’s decision.

The ultimate goal of the analytical pipeline proposed in this paper is providing tools that are both usable and interpretable for the radiology expert in the hope that such interpretation helps revealing new insights about the problem itself. To enhance interpretability and explainability we propose coupling the data transformations on different modalities with feature selection, trying to find an as parsimonious as possible selection of transformed features that accurately distinguishes between treated and control cases. Special focus is placed on the generation of intuitive visual nosological representations^11^ of the analyzed tumors.

## Methods

### Data

The retrospective data analyzed in this study were acquired from 63 mice^6,8,9^, in which tumors were induced by stereotactical injection of GL261 GB cells. Each individual is identified by a unique code (*CXXX*, being *XXX* a sequential number). Mice were divided into control subjects (untreated, $n=29$) and TMZ-treated mice ($n=34$). Out of these, 32 (19 treated, 13 untreated) of them were used to create the classification models, whereas 31 (15 treated, 16 untreated) were used as a holdout sample to gauge the performance of those models (see Ref.^8^ for details on TMZ administration route and schedule). The dataset is further detailed in the supplementary materials. Therapy started eleven days after tumor generation. Tumor volume evolution was followed by MRI and MRSI, both acquired at chosen time points. Depending on the mice, acquisition can be of a single slice or multi-slice, thus the number of MRI images or MRSI grids acquired may vary from one to four in the same axis for each mouse. That entails a big difference in the number of samples among acquisitions and also limits the possibility of applying 3D techniques, due to the absence of several slices in many samples. Mice were euthanized and histopathological validation performed at endpoint, or chosen time points, depending on the case. All studies were approved by the local ethics committee [*Comissió d’Ètica en l’Experimentació Animal i Humana* (CEEAH)], according to the regional and state legislations (protocol CEEAH 3665). All experiments were performed in accordance with national and autonomic regulations regarding animal housing, supervision and welfare.

**Figure 1:**
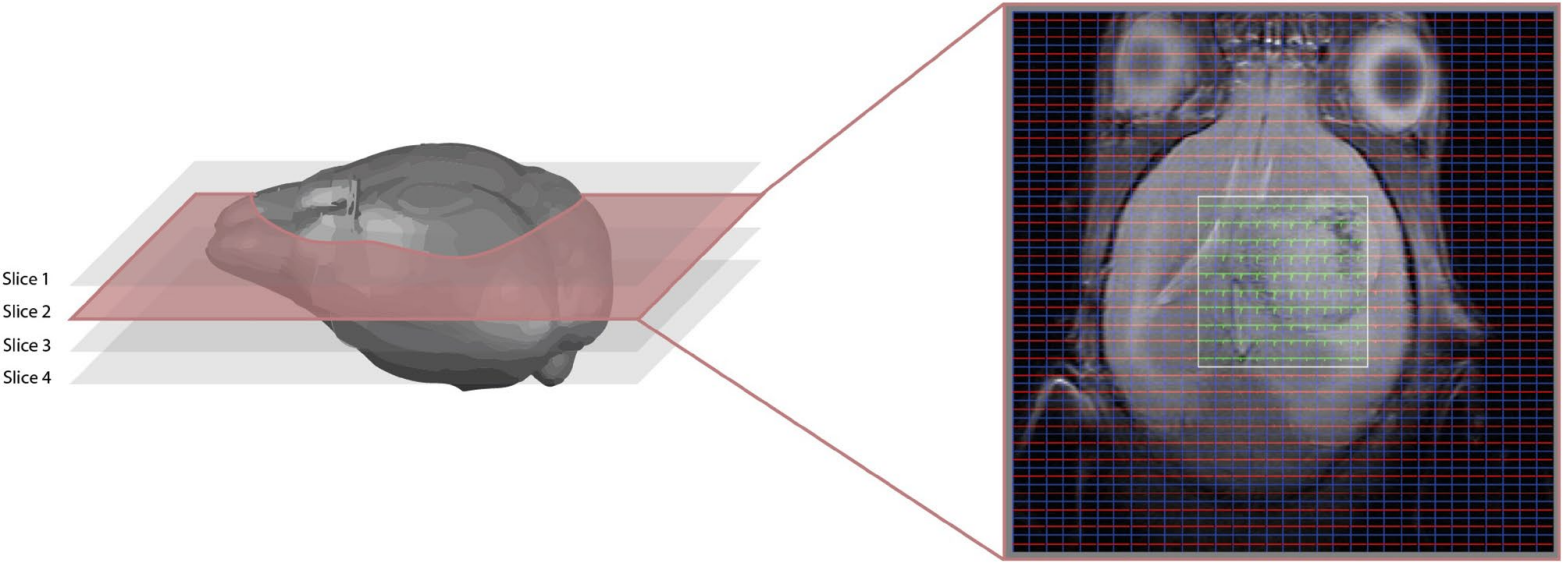
Left, schematic representation of a mouse brain and slice positioning. Right, *T*2*w* MRI image for case C234, with the $32\times 32$ grid superimposed over it. Spectra colored in green are the ones belonging to the VoI (yellow square) of $10\times 10$ or $12\times 12$ enclosing the data used in the study. The rest of spectra are represented in red. Blue lines separate all grid pixels.

The data thus included both MRI and MRSI information. For this work, the available MRI was a *T*2 weighted (*T*2*w*) acquisition of the mouse brain, producing between one and four images of $256\times 256$ pixels. This was the basis for choosing the most suitable zone for MRSI acquisition. The MRSI grid used (which consists of $10\times 10$ or $12\times 12$ voxels that can be treated as *pixels* in the analytical pipeline) can be graphically superimposed to the MRI image, where each element of the grid is associated with one spectrum (Fig. 1). The grid covers mostly the tumor/peritumoral zone, and was used as the input for this part of the study. In the present work, only short echo time (TE) spectra were used and in this sense no negative signals are expected. However, we have chosen to use cNMF in case a similar approach is intented to be used in future work with long TE spectra, and also because the sources can be understood as representative signal centroids. Before applying the designed analytical pipeline, raw spectra were processed with 3D Interactive Chemical Shift Imaging software (version 1.9.11, Columbia University) and normalized with Dynamic MRSI Processing Module (DMPM, GABRMN-*Universitat Autònoma de Barcelona*^12^), while MRI was used without any further transformation.

In summary, for each of the 63 mice, we had between one and four $256\times 256$ MRI images with their corresponding MRSI acquisition slices (1-4 depending on the case), and between 100 and 400 spectra distributed in $10\times 10$ or $12\times 12$ grids, covering most of the tumoral zone.

### Radiomics

Radiomic features are calculated over a whole RoI that can, in principle, be in 2D or 3D^13^. Note that images can indeed be analyzed as such, without transformation. Radiomics feature extraction, though, implicitly generates a data dimensionality reduction that, eventually, retains the predictive capability of the original data. This parsimonious description may provide two inter-related advantages: a less over-fitting prone training sample, especially in low cases-to-features ratio samples such as the one under study, and an easier to interpret data description for radiologists when it comes to explaining the differences between treated and control cases. In this case, as previously mentioned, the treatment schedule used for GL261 GB is known to produce either transient response or cure, so it is reasonable to state that any local changes spotted by MRI/MRSI may be essentially due to response to treatment.

In this study, information to distinguish whether mice belong to the control or TMZ-treated groups comes mostly from the tumor tissue, reducing the region used to extract the Radiomics metrics and thus avoiding the inclusion of peritumoral/normal tissue. To achieve that, manual segmentation of the tumor was performed with expert supervision. Two types of Radiomic features were extracted to amplify the available mineable information: texture features and Minkowski functions.

First, 42 texture features were obtained using Radiomics MATLAB toolbox^14^ (listed in the supplementary materials), which extracts them from four texture matrices, namely: *Gray Level Co-Ocurrence Matrix* (GLCM), *Gray Level Size Zone Matrix* (GLSZM), *Gray Level Run Length Matrix* (GLRLM) and *Neighbouring Gray Tone Difference Matrix* (NGTDM). These matrices offer different useful representations of the texture of an image. In order to calculate them, an initial rectangular matrix is required as input. Since the segmented tumor does not have this shape, an additional step of automatic selection of the smallest rectangle that contains the whole tumor region was applied.

**Figure 2:**
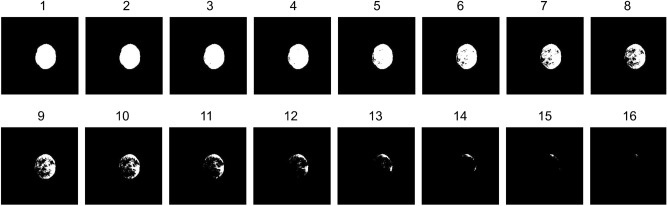
Example of a 16-levels Minkowski thresholds over a tumor mask (C526, treated case, day 18).

Second, Minkowski functions were obtained directly from the segmented tumor mask. These are morphological and structural descriptors of image heterogeneity that can characterize the tumor^15,16^. A number *N* of levels is selected, and *N* auxiliary binary images are created with *N* thresholds that are equally spaced along with the range of intensities of the mask (See example in Fig. 2). The *area*, *perimeter* and *Euler parameter* of the binarized object are then calculated, resulting in 3*N* features. From now on, these features will be referred to as Area#, Perimeter# or Euler#, being # the number of the threshold level associated with that feature. This transformation was performed using a specific MATLAB toolbox^17^ and a range of different values of *N* was explored, settling for a value of 16 and, therefore, generating 48 features. The whole Radiomics set (texture + Minkowski functions) thus consisted of a total of 90 features for every slice.

Data transformation using Radiomics still yields a large number of features that, as previously mentioned, would increase the risk of data over-fitting^7^. One way to decrease such risk is through further dimensionality reduction in the form of feature selection, as described in the following sections.

**Figure 3:**
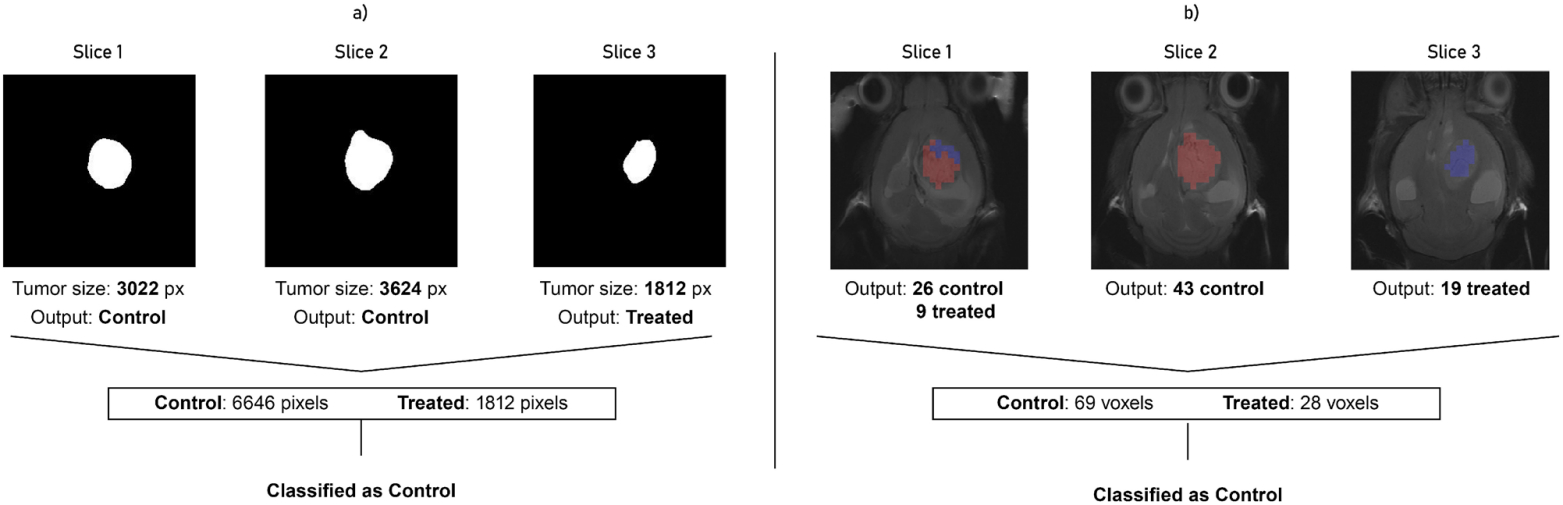
Example of how voting systems work on mouse C1320, control case. (**a**, left) Slice-voting system (SVS); (**b**, right) Voxel-voting system (VVS).

Aiming to imitate a decision-support system, where the final response provided must be a single output for every analyzed subject, in the case of multi-slice mice, it is necessary to gather all the outputs obtained for each slice and combine them into a single result. Keeping that in mind, a *slice-voting* system (SVS for short, hereafter) was implemented, where a weighted average of all the slices responses represents the output for every mouse (see a graphical illustration on the left hand side of Fig. 3). In this voting system, the essence is to offer more decision weight to those slices with a higher amount of tumor represented, thus the weights are based on the number of pixels in the tumor mask used for feature extraction, increasing the importance of central slices and reducing the periphery influence.

### Convex-NMF

Although the use of Radiomics is a suitable approach to tumor characterization, it is not directly applicable to MRSI. This is because MRSI, even if anatomically located, consists of a group of spectral vectors describing signal in the frequency domain, from which texture matrices cannot be extracted. These spectra contain tissue metabolomic information and it can be assumed that, as complex compound signals, they are the result of the combination of different sources (i.e. *paradigmatic* spectra characteristic of certain tissue types of pathological conditions). Accordingly, methods of blind source separation are suitable to extract those sources. They can be seen as feature extractors as well as data dimensionality reductors.

In this study, we used Convex-NMF (non-negative matrix factorization)^18^, an unsupervised method that extracts individual sources from a signal that results from a combination of those sources through a mixing matrix. This variant of NMF just allows non-negative components in both source and mixing matrices, even though it allows negative values in the signal itself.

NMF and Convex-NMF are not new to the neuro-oncology domain, where they have been used to differentiate abnormal masses^19^; to distinguish non-tumoral, responding and non-responding tumoral tissue in glioblastoma through source extraction in a semi-supervised way^20^; for tumor type classification^21^; or for spectral data quality control^22^, to name just a few applications.

**Figure 4:**
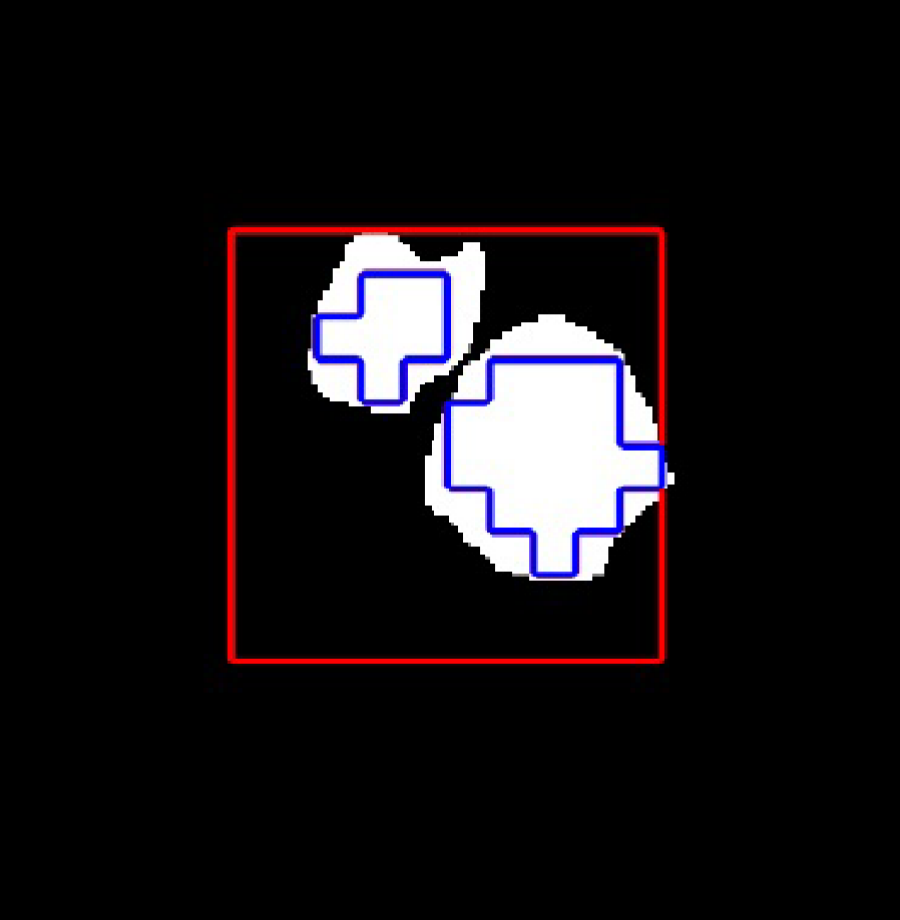
Example of how only spectra regions that are completely inside the tumor (blue outline) are selected from all the grid (red outline). Mouse C234.

Here, as in the Radiomics-based approach, only information extracted from tumor regions was considered, and only spectra that represent completely the tumor region were selected based on the manual segmentation of the tumor (see an example in Fig. 4). This reduces the initial 12,700 available spectra (3200 for model training and 9500 as holdout sample) to a subset of 4465 (1433 for training and 3032 as holdout).

Convex-NMF was applied to the 4465 spectra to extract 20 source vectors. These sources can be qualitatively analyzed by radiology experts in terms of different metabolite contribution. On the other hand, the mixing matrix may inform of how these sources are combined to create each spectrum. Source weights were used as features in the subsequent classification models.

One of the benefits of the grids of MRSI is that they will provide results and information of different anatomical areas inside the tumor, letting us know how heterogeneous they are and how this influences the way each area of the tumor is responding.

Also, as for the Radiomics approach, a method to obtain a single Convex-NMF classification result for each mouse was developed in order to imitate a decision-support system (see an example on the right hand side of Fig. 3). In this case, decision weighting is easier than for MRI, because every single spectrum voxel covers the same amount of tissue, so it is only necessary to count the voxels classified as control in all the slices and compare this with the number of classified as treated. The most abundant one will inform the final model output. This method is the *voxel-voting* system (VVS from now on).

### Feature selection

High data dimensionality increases the risk of model over-fitting, while irrelevant and redundant features increase the search space and make pattern detection difficult, as well as risk obfuscating the classification models. This problem can be alleviated either through an exponential increase of the number of cases in the analyzed sample or through dimensionality reduction methods. Feature selection is an important step to extract more consistent knowledge from our data that are relevant to the problem, discarding redundant features^23^.

Although the choice of the optimal feature selection algorithm is data-dependent, wrapper feature selection methods usually obtain good results, even though at the expense of a high computational cost. In the wrapper scheme, the accuracy of the classifier is used to gauge the relevance of a subset of features, unlike in the filter scheme, where the classifier is built only after the features are already selected. In a third approach, the embedded scheme, the internal structure of the classifier itself allows assigning a measure of relevance to every feature. These three schemes can in turn be applied with different search algorithms (exponential, sequential, etc.). See Ref.^24^ for further details.

In the present study, feature selection was used in two distinct situations. In MRI analysis, which is a low cases-to-features ratio scenario (32-to-90 in the training set), feature selection must be performed in combination with a not-too-complex classifier to ensure model generalization and reliability. This is different from the source extraction analysis in MRSI. In this case, even if the data dimensionality does not force us to apply feature selection, it should lead us to a better quantitative and qualitative understanding of which sources are most responsible for the differentiation of control and treated cases. For these reasons and for the sake of the clinical interpretability of the feature selection results, only linear models were used in both cases.

Similar feature selection approaches were applied to both pipelines. First, a univariate *t*-test allowed to establish a rough rank of their singular importance in the separation of control and treated cases, following a filter scheme^25^. In a second experiment, we applied a recursive feature elimination (RFE) search scheme that starts from the whole set of features and removes one feature (the one considered less relevant) at every step. For the MRSI data, a standard wrapper RFE was applied. For the MRI data, a RFE wrapper did not allow to distinguish the relevance of the features (most accuracies were equal in the first steps) and, therefore, an embedded RFE with linear classifiers was performed instead, and, over the selection of the embedded method, a wrapper feature selection was applied in another step, aiming to reduce even more the size of the subset of selected features. In the embedded RFE scheme, the relevance of a feature was computed as follows: suppose that we have trained *K* linear classifiers with different data partitions (as in a *K*-fold cross-validation), so that every classifier can be expressed as $f_j(x) = g\left( \sum _{i=1}^{N} \omega _{ij} x_i\right)$, where *N* is the number of features and *g* is some monotonic function (such as the linear function or the logistic function for the logistic regression classifier). Then, the relevance of a feature *i* can be expressed as the mean of $\left\{ |\omega _{ij}|\right\} _{j=1}^K$. This idea is based on the hypothesis that irrelevant features produce smaller variations in the output values than relevant ones. Hence, a natural way to compute the relevance of a feature *i* in the trained model $f_j(x)$ is to compute the absolute value of the derivative of $f_j(x)$ with respect to $x_i$, which is $\left| g'\left( \sum _{i=1}^{N} \omega _{ij} x_i\right) \omega _{ij}\right|$. If we only want to compare the relevance between two features, the term $g'\left( \sum _{i=1}^{N} \omega _{ij} x_i\right)$ can be ignored.

### Nosological visualization of the Convex-NMF sources-based classifier

The anatomically-bounded information provided by the cNMF-based prediction allows us to add a layer of information on top of the raw classification itself. Such information will reflect how the responsiveness to TMZ behaves across the tumor structure and *how certain the classifier is about the results yielded for each voxel* (and, therefore, for the different tumor regions). Such certainty is quantified as the posterior probability *P*(*s*) of the label predicted for the input spectra, described as follows:

$$\begin{aligned} P(s_j) = {\left\{ \begin{array}{ll} 0; &{}\quad s_j < \max \limits _{y_k = -1} s_k\\ \pi ; &{}\quad \max \limits _{y_k = -1} s_k \le s_j \le \min \limits _{y_k = +1} s_k\\ 1; &{}\quad s_j > \min \limits _{y_k = +1} \end{array}\right. } \end{aligned}$$

where observation *j* is in class $k = \left\{ -1,1\right\}$; $s_j$ is the score of observation *j*; $+\,1$ and $-\,1$ denote the positive and negative classes, respectively; and $\pi$ is the prior probability that an observation is in the positive class^26^. These nosological maps aim not just to show the certainty of the classifier output, but also to use the map itself as a visual tool for the radiologist to see the response distribution on a subject. They are likely not to show just a binary state (responding–not responding) of the tumor, but, instead, a more nuanced gradation of the response along the anatomical axes.

Overlaying this representation, binary state maps can be created to visually display and identify which individual spatial voxels, or regions of voxels, are misclassified in different slices.

### The analytical pipeline in a nutshell

For the sake of clarity, the dual analytical pipeline is summarized here. For the same cases, we have both MRI and MRSI representations. To address the unbalance in the ratio of cases-to-features in the data set, we apply radiomics to the MRI and cNMF source extraction to the MRSI. To further improve that ratio and to increase the interpretability of the classification results, parallel filter and wrapper feature selection procedures are implemented for both sets of extracted features. Classifiers are implemented and selected, with their evaluation complemented by the use of voting procedures for multi-slice cases (SVS for the radiomic features from MRI and VVS for the features extracted by cNMF from MRSI). Finally, we take advantage of the anatomical coherence of the MRSI to provide an intuitive, radiologist-friendly visualization, using nosological images, of the level of certainty of the classification results.

## Results

The 63 mice of the dataset under analysis were split into two groups, as follows: 32 mice with single-slice MRSI acquisitions were used to train the models, while 31 mice with multi-slice MRSI acquisition (95 MRSI slices in total) were used to test the potential of the classification models over unseen cases. Note that, strictly, the latter is not a test or hold-out set in the common ML sense, because given the restricted number of mice included, there is limited certainty that they can be considered as a representative sample drawn from the same distribution.

The relevance of MRI and MRSI data features for the discrimination between control and treated cases was evaluated using two very distinct approaches. First, they were ranked according to a crude filter feature selection criterion, namely a univariate *t*-test. The second approach attempted to rank features according to a more sophisticated multivariate embedded-wrapper type feature selection procedure, as described in the “Methods” section. Here, the ultimate target is finding the most parsimonious selection of features retaining an optimal discrimination accuracy between control and treated cases.

Embedded-wrapper type feature selection was performed for both MRI and MRSI datasets using different linear classification models, namely logistic regression (LR), linear discriminant analysis (LDA) and support vector machine (SVM) with linear kernel. These models were selected as arguably being the best-known linear classification models and commonly used in ML. Out of these, we report here only the results of the best performers. An LR classifier was selected for the Radiomics features. Importantly, this linear classifier allows a low degree of over-fitting on our limited number of samples. An SVM with linear kernel was selected for the classification of MRSI data, also with quite good results.

**Figure 5:**
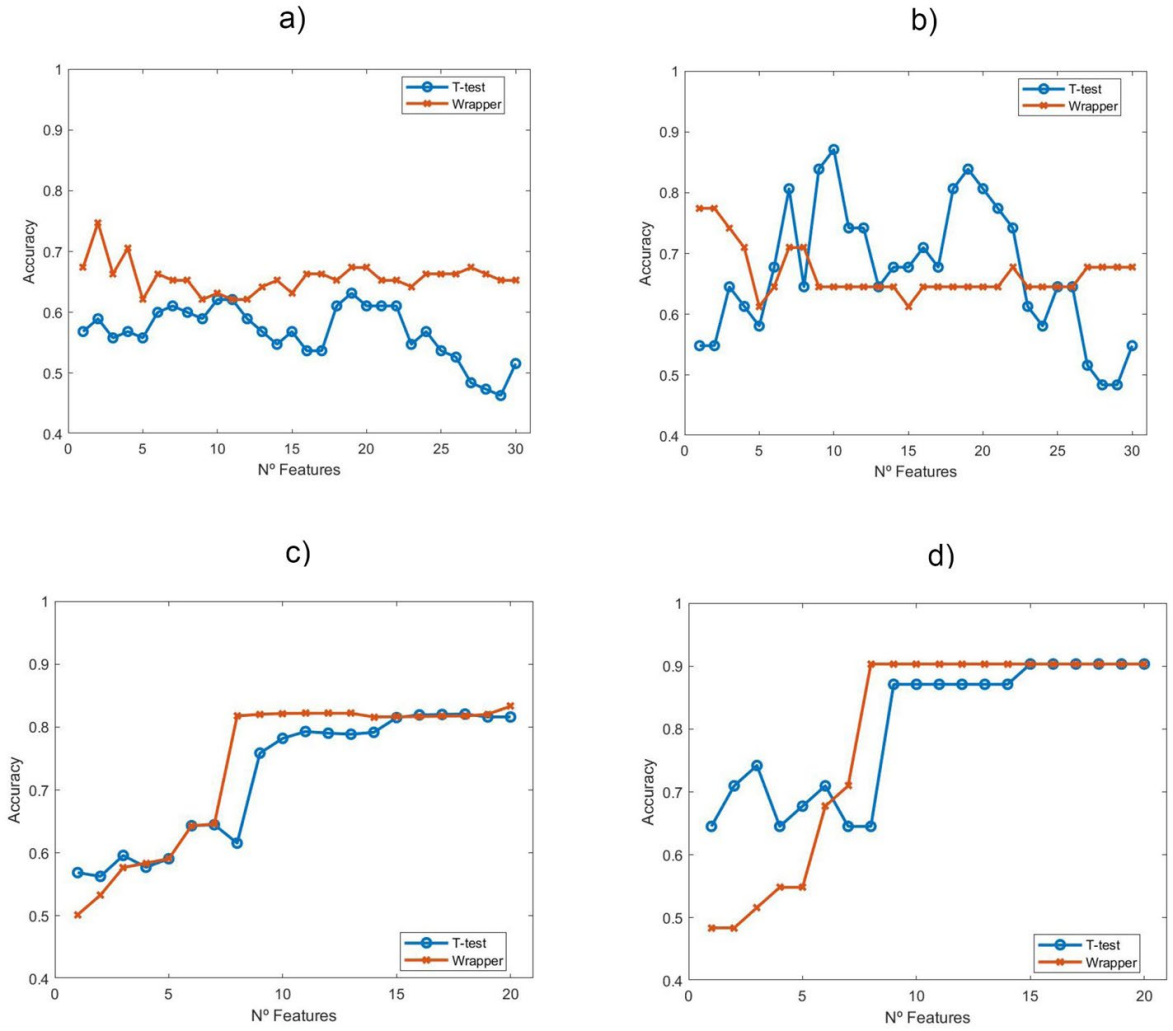
Top: representation of performance (accuracy over the hold-out set) of Logistic Regression over the number of Radiomics features selected (*n* most relevant features according to *t*-test, with $n=1,\ldots ,30$) for MRI data in (**a**) and (**b**). Slice classification method was used in (**a**) whyle SVS method was used in (**b**). Bottom: performance of SVM classifier with linear kernel (accuracy for the hold-out set) over the number of sources selected for the MRSI data in (**c**) and (**d**). Voxel classification method was used in (**c**), while VVS method was used in (**d**).

Regarding the MRI data analysis pipeline, the results for the feature subsets corresponding to the two used approaches, varying from 1 to 30 features, are presented in Fig. 5. For the *t*-test, the features are directly ranked by relevance. In the wrapper approach, instead, each subset corresponds to the best results for that number of features. In both cases, each subset of features is included in the subsets of higher dimensions. This also means that all features except the 30 most relevant were directly discarded for two reasons: first, the low cases-to-features ratio and second, the quest for parsimonious, explainable solutions. Complete rankings of the 90 features for both feature selection approaches are shown in supplementary materials.

In the MRSI pipeline, all 20 extracted sources were considered, and the classification performance results are graphically depicted in Fig. 5. In supplementary materials, the full set of 20 sources is shown with an ID number and both rankings are presented to inform of the relevance of each source.

Note that these figures report the accuracy of the different classifiers over the hold-out set after a training procedure. This accuracy is calculated according to both the SVS and VVS for each classifier. Leave-one-out cross-validation was used in the Radiomics setting due to the low number of samples, whereas in source extraction, where samples are more abundant, 20-fold cross-validation was applied.

**Figure 6:**
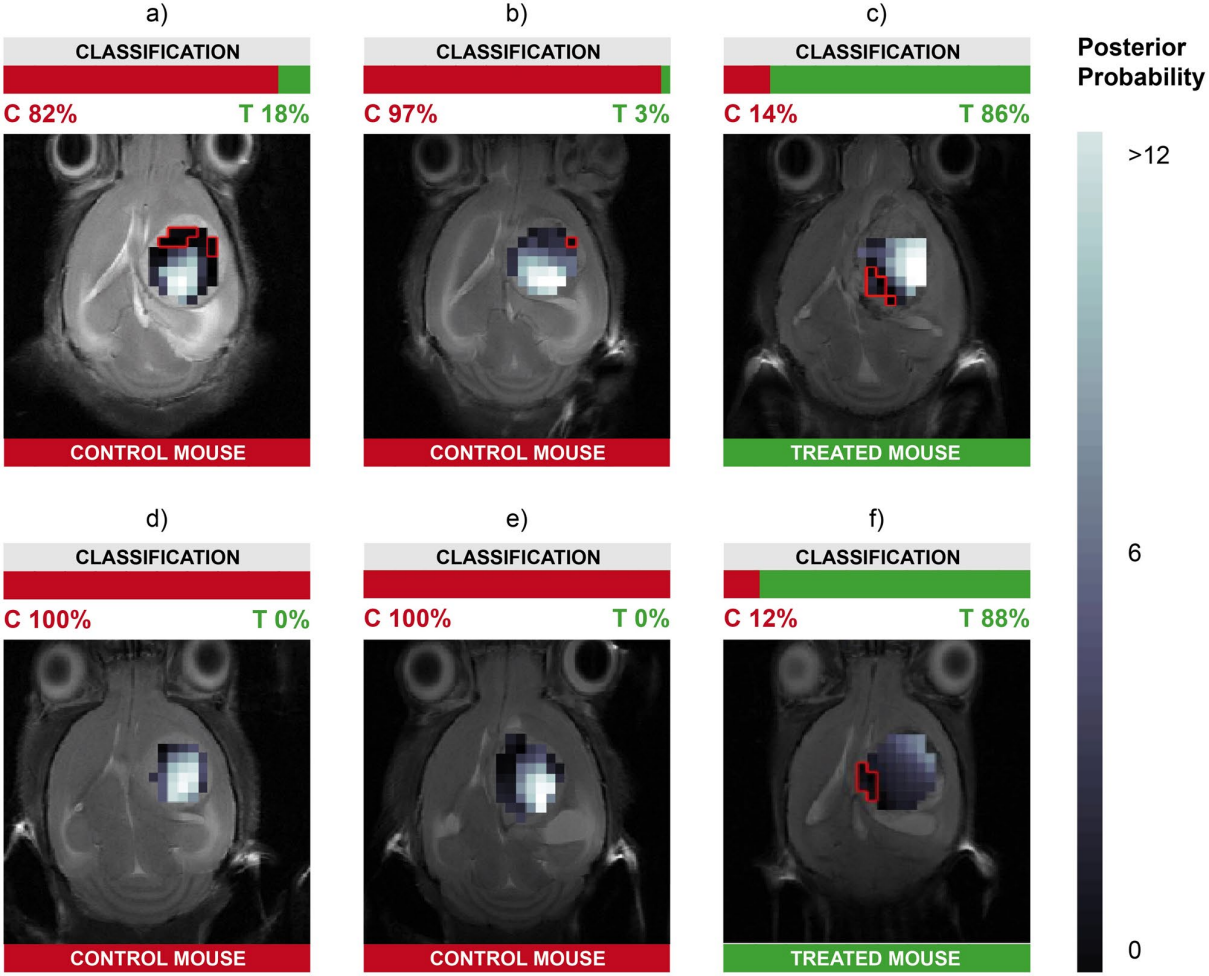
Examples of nosological visual representation of the classification results for MRSI data from their extracted sources. They are meant to provide radiologists with an intuitive interpretation tool. They consist of horizontal T2w MRI images of GL261 GB afflicted mice at different treatment/evolution day, superimposed with representative nosological maps of the classification reliability in different tumor regions for the SVM classifier with linear kernel and 10 features (sources) selected by t-test. The color-coding (see scale on the right) shows how reliable the model classification output can be considered and it represents a classifier output posterior probability. The lighter the color, the more reliable and *vice versa*. The red contour over some of the voxels represents those that were misclassified by that model. (**a**) C1465-day15, (**b**) C1109-day11, (**c**) C1412-day23, (**d**) C1474-day14, (**e**) C1320-day18, (**f**) C1026-day23. The color bars at the bottom represent the true class of the case, whereas the color bars at the top represent the percentage of voxels classified either as treated or as control for each case.

The anatomically-grounded visual representation of the proposed analytical pipeline is exemplified by the nosological MRI-based images in Fig. 6, which simultaneously display the classification boundaries and the degree of certainty of the classification outcome (results for all mice and classifiers can again be found in the supplementary materials).

## Discussion

### Radiomics study

The study of tissue morphology is used in oncology to detect the presence of disease and also to check tissue local changes determined by treatment.

Qualitative neuroimaging analysis is usually hampered by a lack of reproducible and objective measures, although approaches such as the VASARI features have been proposed^27^. For this reason, quantitative assessment of tissue morphology as part of an at least semi-automated analytical pipeline could be of great practical interest. MRI-based studies using Radiomics quantitative descriptions of MRI images have been found to predict brain tumor survival/progression, or follow response to therapy^7,15,28,29^. Radiomics is an emerging technique used in radiology that aims at extracting quantitative information features from medical images that potentially bear descriptive and predictive capabilities^13^. This type of feature extraction or transformation is able to predict characteristics of different types of diseases and has already been applied to the analysis of GB images for survival prediction and patient stratification^30^.

The results reported in Fig. 5 show that Radiomic features can discriminate fairly well between treated and control cases in our preclinical study. LR results show a somehow irregular pattern that overall deteriorates as the number of patterns increase. This is not unexpected, due to the small sample size. In the wrapper approach, over 75% hold-out accuracy can be achieved with just two features (namely *Gray-Level Co-Occurrence Matrix (GLCM) Entropy* and *Perimeter9*), both with the slice classification and SVS methods. The *t*-test ranking, though, nears 90% accuracy for 10 features (the first 10 listed in the ranking of Table S3 in the supplementary materials) with the SVS method.

**Figure 7:**
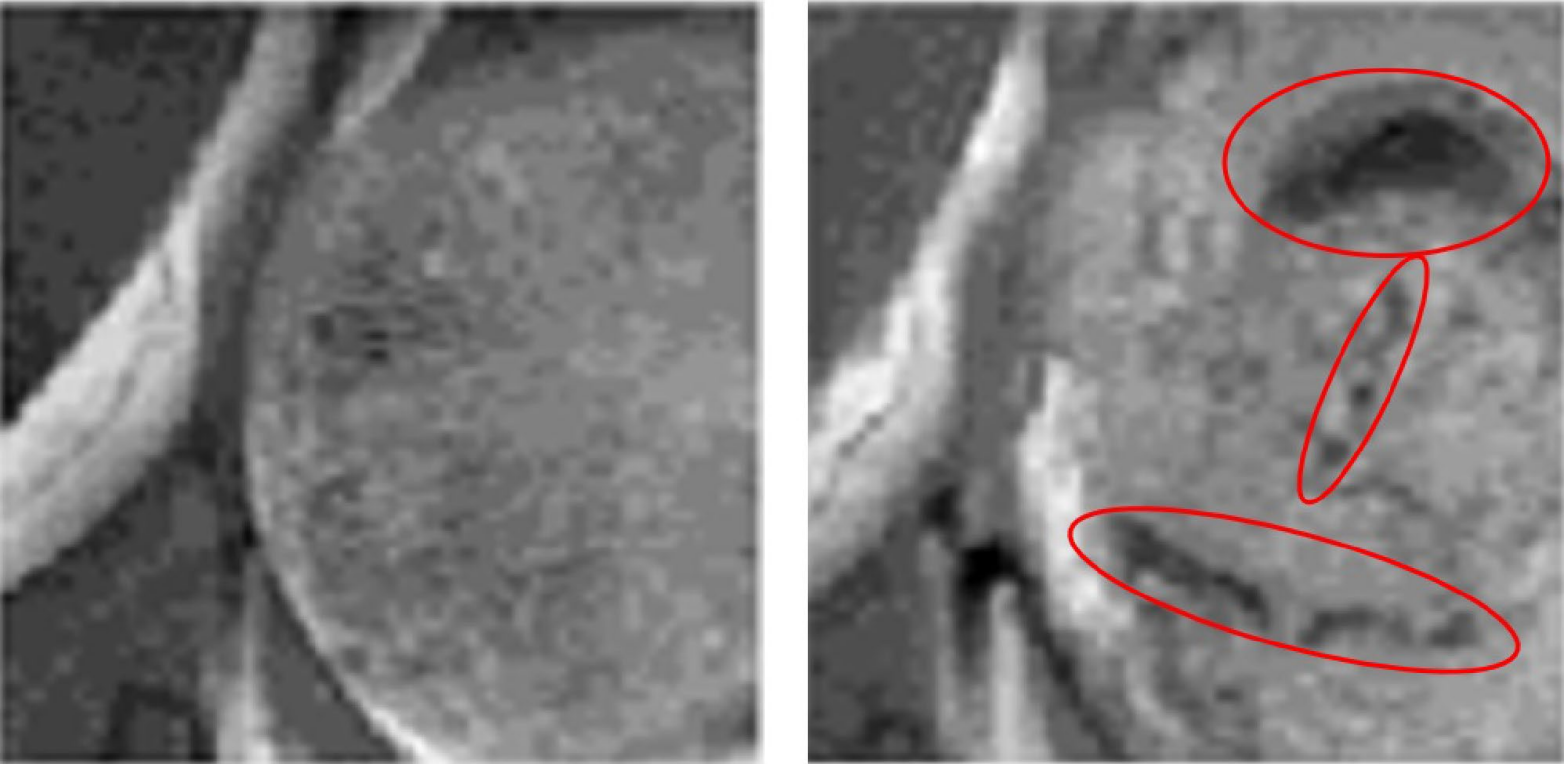
Examples of control (left, mouse C583) and treated, transiently responding to TMZ according to histopathological parameters (right, mouse C574) murine GL261 GB tumors. Note the appearance of hypointense zones (red circles) in T2w MRI from the treated mouse, noticeably different from the more homogeneous appearance observed in the control case.

Malignant brain tumors usually have a heterogeneous appearance in MRI, due to the presence of necrosis and/or hemorrhagic foci. Moreover, the architecture of the tissue can change upon treatment^31,32^; at the histopathological level, transient response to TMZ, detected in our preclinical GB model under different protocols, leads to the appearance of giant cells, decrease of the proliferative rate and increase of acellular spaces^9^. Altogether, this may have a clear impact on MRI and its associated features (see MRI examples of treated and control mice in Fig. 7).

Moreover, changes in tumor properties such as ischemia, angioedema and avascular necrosis, might be more obvious on *T*2*w* MRI, reinforcing the potential of this approach alone for a radiomics-based therapy response analysis. On the other hand, Minkowski functions have been described as useful in analyzing heterogeneity of peritumoral hyperintensity of glioblastoma, demonstrating prognostic value in predicting survival^33^. Our results are supported by findings reported by other authors: GLCM, which is a method that describes how similar neighboring voxels are by their gray values, was determinant to distinguish between progression and pseudoprogression in human GB patients treated with the current standard treatment, spotting tumors that were truly responding to therapy^34^. Other authors have used GLCMs in order to distinguish GBM phenotypes (i.e. Necrosis, Active Tumor, Edema)^35^, while authors in^36^ have used texture features such as GLCM to study local and regional heterogeneity and their role in the survival stratification of patients with GB. Other radiomic variables such as GLRLM and GLSZM also ranked in high positions in this work. This agrees with the available literature, for example, Ref.^37^ describes features such as GLRLM correlating with histopathological features such as Ki67 in high-grade glioma. Recent work^38^ also described the value of GLRLM and GLSZM for evaluating response to therapy in GB, being able to distinguish pseudoprogression from true progression.

Recent results^6^ suggest that treated GL261 tumors responding to TMZ treatment present significantly higher immune system elements within the tumor region. The local effects of these cells against the GB mass may also cause local changes that could be spotted by MRI, although further work would be needed to establish a proper correlation between MRI features and immune system elements presence/action. Among cases that had poor classification in radiomics approach, we find mice C975 and C1451 (both treated cases misclassified as controls with the radiomics approach). These cases proved to have mixed heterogeneous pattern of response/no response when studied with semi-supervised approaches as in^8^ and case C975 presented only a slight response to TMZ administration. In this sense, it is not surprising its misclassification with radiomics as well, since local tissue effects may have a clear impact on MRI features. In this respect, it is wise to mention here that MRSI features detected from treated GL261 mice have been found to oscillate along time^6,9^, with a ca. 6-day period. This longitudinal heterogeneity, unless taken into account in the analysis, may cause errors in the classification due to the use of single time point data. Case C1456 was a treated case misclasified as a control both in radiomics and MRSI-based approaches. This case had no remarkable features that could justify such misclassification, except for being a slow-growing case, with a borderline tumor volume at the time of examination, which could point to a limit in the sensibility of different approaches when the studied zone, and, accordingly, the available information decreases significantly.

One of the challenges still hampering proper comparison between different Radiomics-based studies is the lack of standardization^39^. Due to the well known contrast-enhancement characteristics of GB tumors, some authors emphasize the potential of contrast-based imaging modalities^29^, but since contrast agents are not recommended for some patients (e.g. with kidney diseases), a reliable method based on *T*2*w* MRI would be of great interest for its translational potential.

### MRSI study

**Figure 8:**
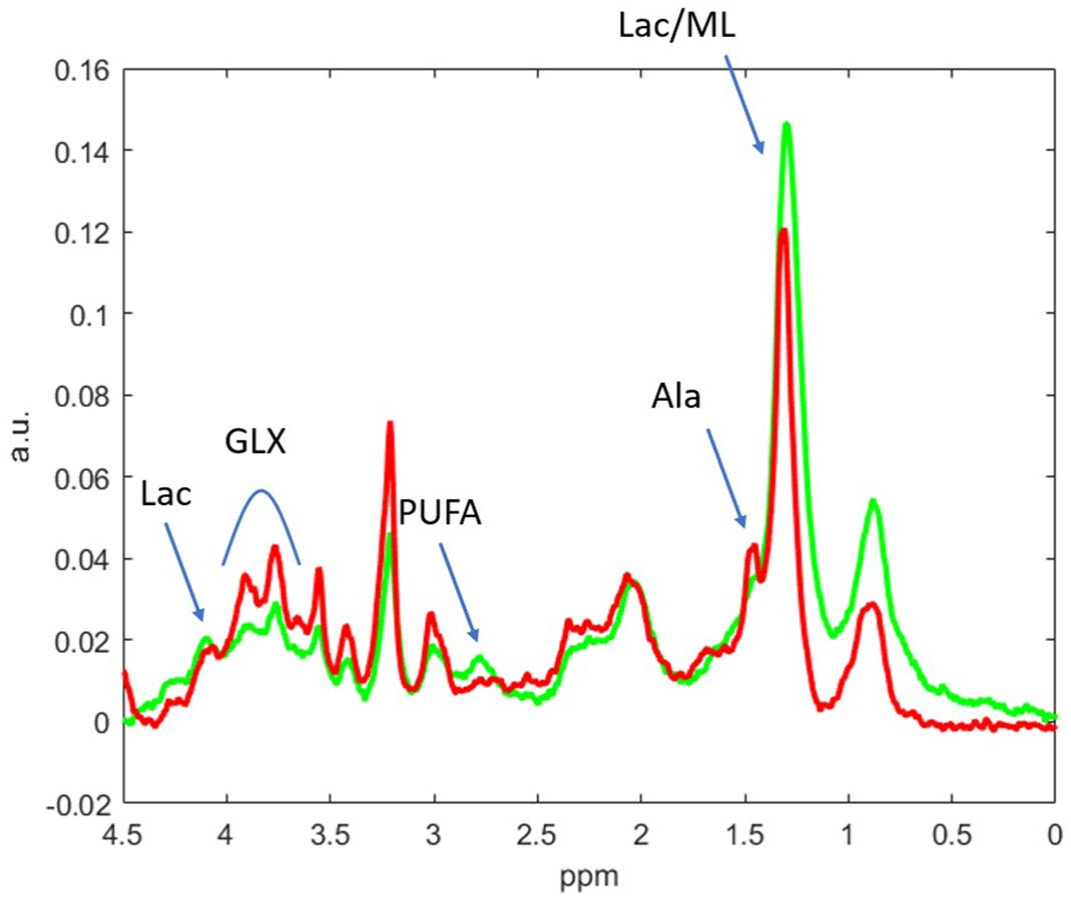
Examples of two selected sources relevant for distinguishing spectra from control and treated, responding murine GL261 GB tumors. Some relevant metabolites have been indicated [Polyunsaturated fatty acids (PUFA), Lactate (Lac), Alanine (Ala) and the signals overlapped from Glutamate-glutamine, alanine and glycine (labeled as Glx)]. The green source shows some of the typical features seen in MRSI pattern of treated, responding tumors such as visible PUFA and relative increase in Lac, while the red source did not show (or only barely visible) PUFA, in addition to increased Glx signals and a more clear Ala presence in comparison with the green source.

The results reported in Fig. 5 for the MRSI data show us two things. First, that they are overall better than those achieved by the best model (LR) applied to the radiomic features; and, second, that they are far more stable in their evolution over the selection of features (sources in this case). The results with the *t*-test selection begin deteriorating faster than with the wrapper approach. The wrapper is able to keep accuracy over 80% in the voxel classification method and over 90% in the VVS method with as little as 8 sources (two of them are shown in Fig. 8, for illustration). With less than 8 sources, the performance quickly deteriorates, meaning that the removal of any of these 8 sources has a significantly negative impact on the discrimination between treated and control cases, but also meaning that any further addition of sources does not benefit such discrimination.

The aforementioned sources represented in Fig. 8 show the expected changes in metabolites previously described in^8^ as relevant for distinguishing among control and responding GL261 murine GB, such as poliunsaturated fatty acids (PUFA), lactate (Lac), glutamate-glutamine (Glx) and alanine (Ala). It is worth noting that the appearance of PUFAs after preclinical brain tumor treatment has been described by other authors such as Refs.^40,41^, probably reflecting local apoptosis as a consequence of therapeutic protocols. Authors in Ref.^41^ also describe Alanine as a distinctive trait between untreated and treated mice. It is also worth mentioning that such metabolomic pattern changes have been also seen during transient responses to a variable set of therapeutic approaches: TMZ in standard or metronomic schedules^9^, CPA^6^, immunotherapy alone or in combination^42^, hinting that changes are not restricted to TMZ treatment.

Metabolomic studies based in MRS/MRSI also proved useful when assessing GB therapy response in clinical settings. Namely, Lactate/Lipids were found useful for differentiation between GB relapse and pseudoprogression^43^ and estimation of overall survival in GB after treatment^44^. The glutamate/glutamine region (Glx) was also studied as a potential therapy response biomarker^45^. PUFAs were also reported as an apoptosis biomarker with in vivo MRS^46^. Overall, data seems to point that metabolomics studies are indeed relevant in therapy response studies, but most of the reported work in therapy response assessment is focused on few metabolites or metabolite ratios. The novelty provided by the pattern recognition studies is the consideration of the whole pattern changes.

All in all, this means that, for the proposed pipeline, we favour the use of the combination of an SVM classifier and the wrapper feature selection method, on the basis of MRSI data, and where results of the Radiomics approach only add useful knowledge in terms of the selection of the most discriminating features of the image.

Most importantly, one of the benefits from the MRSI per-voxel analysis is the possibility to ascertain how each part of the tumor is responding, allowing us to unravel its heterogeneity. Even if considering a mouse as responding to a specific therapy as a whole, we still cannot be sure whether the tumor is consistently and homogeneously responding to therapy or not. The key advantage of our analytical pipeline is that the classification results come with a quantification of the certainty of the classification prediction that is *anatomically bounded*. This means that we can graphically represent, using nosological images, such level of certainty over the anatomy of the tumor, as exemplified by the images in Fig. 6. These images include not only the nosological representation of the level of certainty of the classifier prediction, together with the highlighting of the misclassified voxels, but also the information about the true and predicted classes. The latter allows the expert to know whether the case is treated or control and whether the classifier prediction is treated or control using the VVS method. Merging the information about classifier case prediction with the anatomically-distributed information about such classification certainty should create the type of knowledge extraction synergy that only visualizations can provide^47^, making it an interpretability-enhancing tool at medical expert disposal. These nosological maps can intuitively be interpreted and explained by a trained radiologist, even if not familiarized with MRSI interpretation. They are the cornerstone of the analytical pipeline, to which we can add the knowledge provided by the overall classification itself and the feature relevance (Radiomics and sources) results.

Some of the results found in these prediction reliability maps are striking. Crucially, the voxels misclassified by our models are not distributed individually and randomly all over the tumor, but mostly grouped in contiguous, compact regions; they are also to be found mostly in the external region of the tumor and, furthermore, they consistently show a lower reliability score than the average for that zone. Still, we cannot discard that part of this lack of reliability would be due to poorer homogeneity in spectra from edges of the MRSI grid, in which several of these voxels are located. This reliability score issue is telling us that, even if classification is binary in nature, our misclassified zones are, in general, less certain that the correctly classified cases. This could be interpreted in the sense that a voxel that is not classified as responding to treatment, does not necessarily fully fit the non-responding pattern, which could mean that that region of the tumor might still be responding, but to a lower extent than other regions, or other mice.

Moreover, regarding the misclassified voxels reported in Fig. 6, case C1026 is worth singling out for discussion. This case, also reported in Ref.^9^, proved to be a heterogeneous case with a mix of responding/unresponsive pattern in MRSI, and also presented unexpectedly high Ki67 immunostaining values considering that it was a TMZ-treated case in transient growth arrest. Therefore, it is not surprising that some zones are misclassified with the approach described in this work.

Also, it should be mentioned that two subjects (C1110 and C1111) have shown abnormal behaviour in all the sources-based classifiers, being consistently misclassified with high-reliability scores. These cases were checked manually looking for any artifact or abnormality in their data, but nothing was found. Given the reduced sample size under analysis, this situation negatively impacts classification scores, which indicates the importance of conducting additional studies including more mice.

Last but not least, our preliminary data show that timing is a relevant factor while evaluating tumour response to therapy^6,9^, and our time points were validated by MRSI, growth arrest and, in some cases, histopathological data. Since MRSI can provide earlier data and inform about tumour relapse before tumour volume changes^8^, this may be used in future studies to improve MRI information and refine prediction of whether a tumour is properly responding to therapy or will relapse in a near future. The applicability of this methodology to other clinically relevant treatment approaches in which immune response may be also involved, such as radiotherapy, may be tackled in future studies.

### Conclusions

Both Radiomics and source extraction show potential in therapy response assessment, with advantages and limitations. Source extraction over MRSI offers a higher accuracy and very good anatomical detail that helps extracting knowledge about the tumor behaviour, but its reproducibility over new cases is limited because the weights used are based on sources extracted previously, which means that the whole process should be repeated to analyze new cases.

MRI did not show such level of successful results but, on the other hand, since only a *T*2*w* MRI is enough for proper classification, it would be extremely reproducible along different clinical centers if a future clinical application is launched. Still, all clinical centers are able to perform *T*2*w* MRI acquisitions, while MRSI is not fully incorporated in the clinical pipeline, either due to software issues and challenges in processing and interpretation. Further studies may consider the joint MRI/MRSI categorization, and to search for correlations in MRI aided/guided by MRSI rich information.

The analytical pipeline presented in this paper has led us to a very compact and intuitive visualization of results that summarizes, in an anatomically recognizable representation, a complex combination of feature selection and classification based on MRI Radiomics and MRSI, together with prediction certainty. The ultimate goal is making this visual representation not just a summarization of results, but also a source of inductive reasoning for medical experts, as a tool that may lead to the generation of new hypothesis for the problem of response to therapy for glioblastoma.

## Supplementary information

Supplementary material 1

## Data Availability

MRI and MRSI data files from mice will be made publicly available from the UAB digital repository database, accessible from URL: https://ddd.uab.cat.
