# Supplementary material for: Unraveling response to temozolomide in preclinical GL261 glioblastoma with MRI/MRSI using radiomics and signal source extraction
Source: Sci Rep. 2020 Nov 12;10:19699. doi: 10.1038/s41598-020-76686-y (PMC7661707; doi:10.1038/s41598-020-76686-y)
Supplement: Supplementary file 1 — Supplementary material 1 [file 41598_2020_76686_MOESM1_ESM.pdf]

Unraveling response to Temozolomide in preclinical GL261 glioblastoma with MRI/MRSI using radiomics and signal source extraction

Luis Miguel Núñez, Enrique Romero, Margarida Julià-Sapé, Andrés Santos, Maria Jesús Ledesma-Carbayo, Carles Arús, Ana Paula Candiota and Alfredo Vellido

SUPPLEMENTARY MATERIAL

DATASET

In ***Table S1***, the whole dataset is listed, including relevant characteristics in the classification problem for each mouse.

DATASET							
TRAIN				HOLD-OUT			
MOUSE #	DAY	Number of SLICES	CONTROL/ TREATED	MOUSE #	DAY	Number of SLICES	CONTROL/ TREATED
179	17	1	CONTROL	1109	11	4	CONTROL
233	17	1	CONTROL	1110	13	4	CONTROL
234	17	1	CONTROL	1111	16	4	CONTROL
255	14	1	CONTROL	1112	13	4	CONTROL
278	19	1	CONTROL	1471	16	2	CONTROL
288	18	1	CONTROL	1465	15	2	CONTROL
32	16	1	CONTROL	1472	13	3	CONTROL
351	13	1	CONTROL	1474	14	3	CONTROL
520	18	1	CONTROL	1320	18	3	CONTROL
529	18	1	CONTROL	1344	17	3	CONTROL
583	18	1	CONTROL	1348	21	3	CONTROL
69	15	1	CONTROL	1457	23	3	CONTROL
71	16	1	CONTROL	1459	15	2	CONTROL
526	18	1	TREATED	1461	15	3	CONTROL
572	18	1	TREATED	1462	15	3	CONTROL
574	26	1	TREATED	1466	23	3	CONTROL
776	34	1	TREATED	975	26	4	TREATED
795	18	1	TREATED	1023	23	4	TREATED
797	22	1	TREATED	1026	23	4	TREATED
808	33	1	TREATED	1108	29	3	TREATED
415	22	1	TREATED	1100	26	4	TREATED
418	22	1	TREATED	971	26	4	TREATED
437	23	1	TREATED	1412	23	2	TREATED
525	22	1	TREATED	1445	23	4	TREATED

527	22	1	TREATED	1447	28	3	TREATED
575	26	1	TREATED	1450	24	2	TREATED
584	26	1	TREATED	1451	23	3	TREATED
586	22	1	TREATED	1456	23	2	TREATED
821	34	1	TREATED	1458	23	4	TREATED
414	24	1	TREATED	1460	23	2	TREATED
419	24	1	TREATED	1463	23	1	TREATED
521	18	1	TREATED				

Table S1: Mice dataset details.

RADIOMICS TEXTURE FEATURES

The 42 texture features used in the Radiomics approach of our research are listed in ***Table S2***, according to which matrix they are extracted from.

**Gray Level Co-occurrence Matrix (GLCM)**	
1	Energy
2	Contrast
3	Entropy
4	Homogeneity
5	Correlation
6	SumAverage
7	Variance
8	Dissimilarity
9	Autocorrelation
**Gray Level Run Length Matrix (GLRLM)**	
1	Short Run Emphasis (SRE)
2	Long Run Emphasis (LRE)
3	Gray Level Non-Uniformity (GLN)
4	Run Length Non-Uniformity (RLN)
5	Run Percentage (RP)
6	Low Gray-Level Run Emphasis (LGRE)
7	High Gray-Level Run Emphasis (HGRE)
8	Short Run Low Gray-Level Emphasis (SRLGE)
9	Short Run High Gray-Level Emphasis (SRHGE)
10	Long Run Low Gray-Level Emphasis (LRLGE)
11	Long Run High Gray-Level Emphasis (LRHGE)
12	Gray-Level Variance (GLV)
**Gray-Level Size Zone Matrix (GLSZM):**	
1	Small Zone Emphasis (SZE)
2	Large Zone Emphasis (LZE)
3	Gray-Level Nonuniformity (GLN)
4	Zone-Size Nonuniformity (ZSN)
5	Zone Percentage (ZP)
6	Low Gray-Level Zone Emphasis (LGZE)
7	High Gray-Level Zone Emphasis (HGZE)

8	Small Zone Low Gray-Level Emphasis (SZLGE)
9	Small Zone High Gray-Level Emphasis (SZHGE)
10	Large Zone Low Gray-Level Emphasis (LZLGE)
11	Large Zone High Gray-Level Emphasis (LZHGE)
12	Gray-Level Variance (GLV)
**Neighborhood Gray-Tone Difference Matrix (NGTDM):**	
1	Coarseness
2	Contrast
3	Busyness
4	Complexity
5	Strength

Table S2: Details of radiomics texture features.

RADIOMICS FEATURE SELECTION

Table S3 shows how features were ranked by both by the *t*-test and the wrapper feature selection methods.

Ranking Position	*t*-Test method	Wrapper Method
**1**	**Perimeter9**	**GLCMEntropy**
**2**	**Perimeter8**	**Perimeter9**
**3**	**GLRLMRLV**	**GTDMComplexity**
**4**	**Perimeter7**	**GLSZMSZLGE**
**5**	**Euler7**	**Area16**
**6**	**Euler6**	**Area13**
**7**	**GLRLMGLN**	**GLRLMRLV**
**8**	**Perimeter6**	**GLRLMRLN**
**9**	**GLCMVariance**	**Euler1**
**10**	**GLSZMGLN**	**GLRLMSRE**
**11**	**GLCMEntropy**	**Euler16**
**12**	**Perimeter10**	**GLSZMLGZE**
**13**	**GLCMEnergy**	**Euler15**
**14**	**GLRLMGLNN**	**GLSZMGLV**
**15**	**Euler5**	**GLCMCorrelation**
**16**	**Perimeter5**	**Euler9**
**17**	**GLRLMSRHGE**	**Perimeter16**
**18**	**Euler12**	**Euler2**
**19**	**Euler2**	**Area12**
**20**	**GTDMContrast**	**GLSZMZP**
**21**	**GLSZMSZLGE**	**GTDMStrength**
**22**	**Euler3**	**Area10**
**23**	**Perimeter4**	**GLRLMSRHGE**
**24**	**Euler10**	**GLCMVariance**
**25**	**GLCMCorrelation**	**Euler5**

**26**	**GLSZMLGZE**	**GLCMHomogeneity**
**27**	**GLSZMSZHGE**	**Area11**
**28**	**Euler4**	**GLRLMSRLGE**
**29**	**GLRLMHGRE**	**Perimeter6**
**30**	**GLCMAutoCorrelation**	**GLRLMRP**
31	GLSZMHGZE	GLSZMSZE
32	Euler8	Area14
33	GLCMSumAverage	GTDMContrast
34	Euler11	GLRLMGLNN
35	Perimeter3	Euler14
36	Euler14	Euler10
37	Area16	Euler13
38	GLSZMZSV	GLRLMLGRE
39	Perimeter13	GLRLMLRHGE
40	GLRLMSRE	GLSZMGLN
41	Area12	GLSZMZSN
42	GLRLMRLN	GLSZMLZE
43	GLSZMGLV	Perimeter14
44	GLCMHomogeneity	Perimeter5
45	Perimeter11	Euler11
46	GLSZMZP	Perimeter10
47	Perimeter16	GLCMDissimilarity
48	GTDMStrength	GLCMEnergy
49	GLRLMRP	GLSZMHGZE
50	Euler16	Perimeter7
51	Area13	GLSZMLZLGE
52	Area1	GLRLMGLN
53	Area2	Euler6
54	Area3	GLRLMGLV
55	Perimeter2	Perimeter3
56	Area4	Perimeter11
57	Area5	GLCMSumAverage
58	Euler1	GLRLMLRE
59	GLCMDissimilarity	Perimeter4
60	GLRLMLRE	Area9
61	GLRLMLGRE	Euler7
62	Perimeter1	Euler3
63	Area6	Perimeter2
64	GLRLMLRLGE	Perimeter13
65	GLRLMSRLGE	Perimeter1
66	GLSZMLZE	Euler4
67	Area7	Perimeter12
68	GLSZMLZLGE	GLSZMZSV
69	Perimeter14	GTDMCoarseness
70	GLRLMRLNN	GLRLMRLNN
71	Area8	GLRLMLRLGE

72	GLCMContrast	GLSZMSZHGE
73	GLSZMLZHGE	Euler12
74	Area11	Perimeter8
75	GLRLMLRHGE	GLRLMHGRE
76	Euler9	GLSZMLZHGE
77	Area9	Area1
78	GTDMBusyness	Euler8
79	Area14	Area2
80	Euler15	Area3
81	Area15	Area4
82	GTDMCoarseness	GTDMBusyness
83	GLSZMZSN	Area5
84	GTDMComplexity	Area15
85	Perimeter15	GLCMContrast
86	GLSZMSZE	Area6
87	Area10	Perimeter15
88	GLRLMGLV	GLCMAutoCorrelation
89	Perimeter12	Area7
90	Euler13	Area8

Table S3: Radiomics feature ranking obtained by both selection methods. In bold, the first 30 features used in the paper experiments.

EXTRACTED SOURCES

In the following *Figure S1* the 20 sources extracted from the 4,465 mice spectra are represented. These sources were the ones used in the weights calculation process. Those weights were later used in the whole pipeline for classifiers creation and maps drawing. All the sources have an ID that identifies them and let the corresponding rankings of the *t*-test and wrapper shown in *Table S4* be easily interpretable.

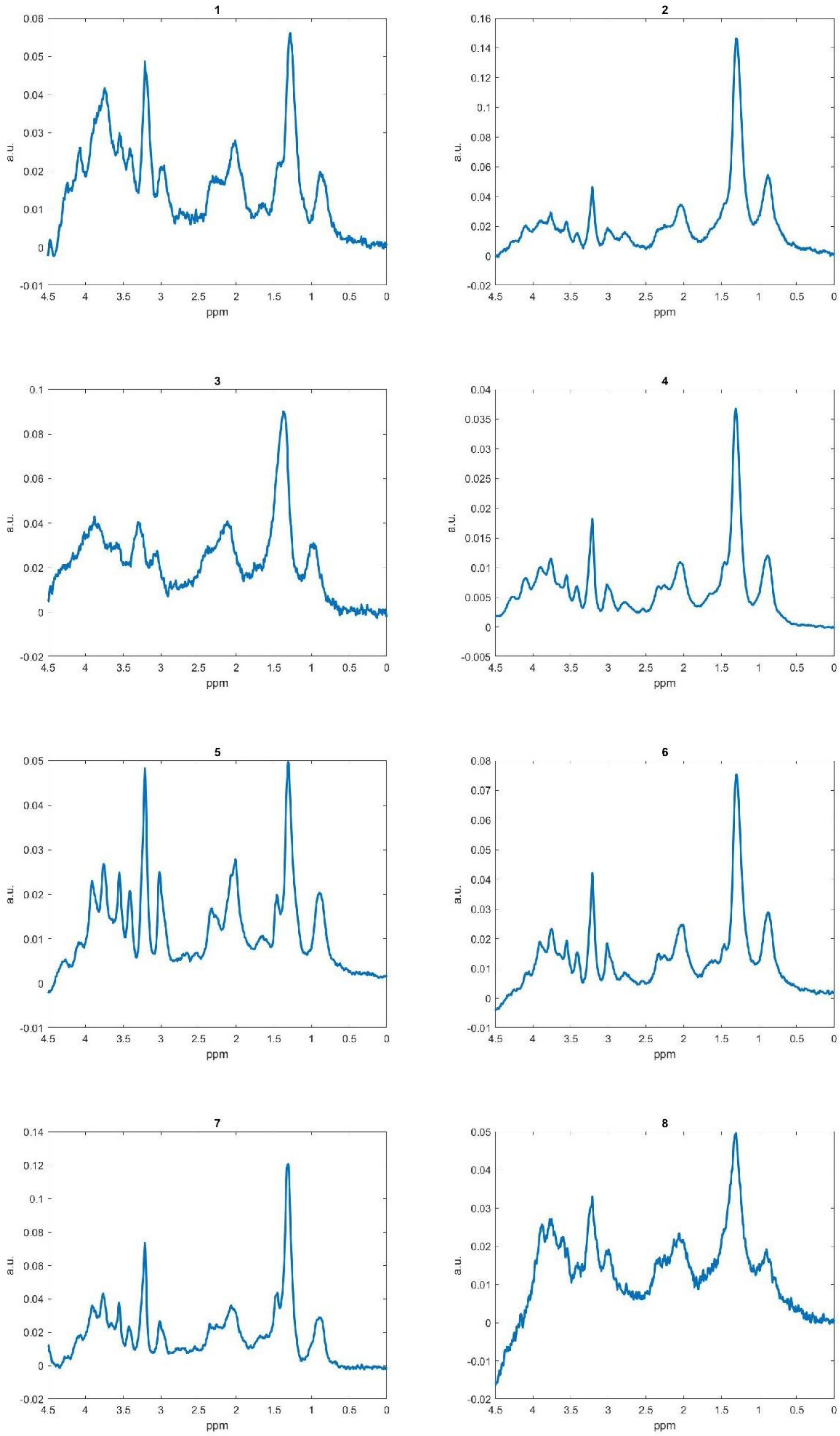
1
2
3
4
5
6
7
8
a.u.
ppm
4.5 4 3.5 3 2.5 2 1.5 1 0.5 0

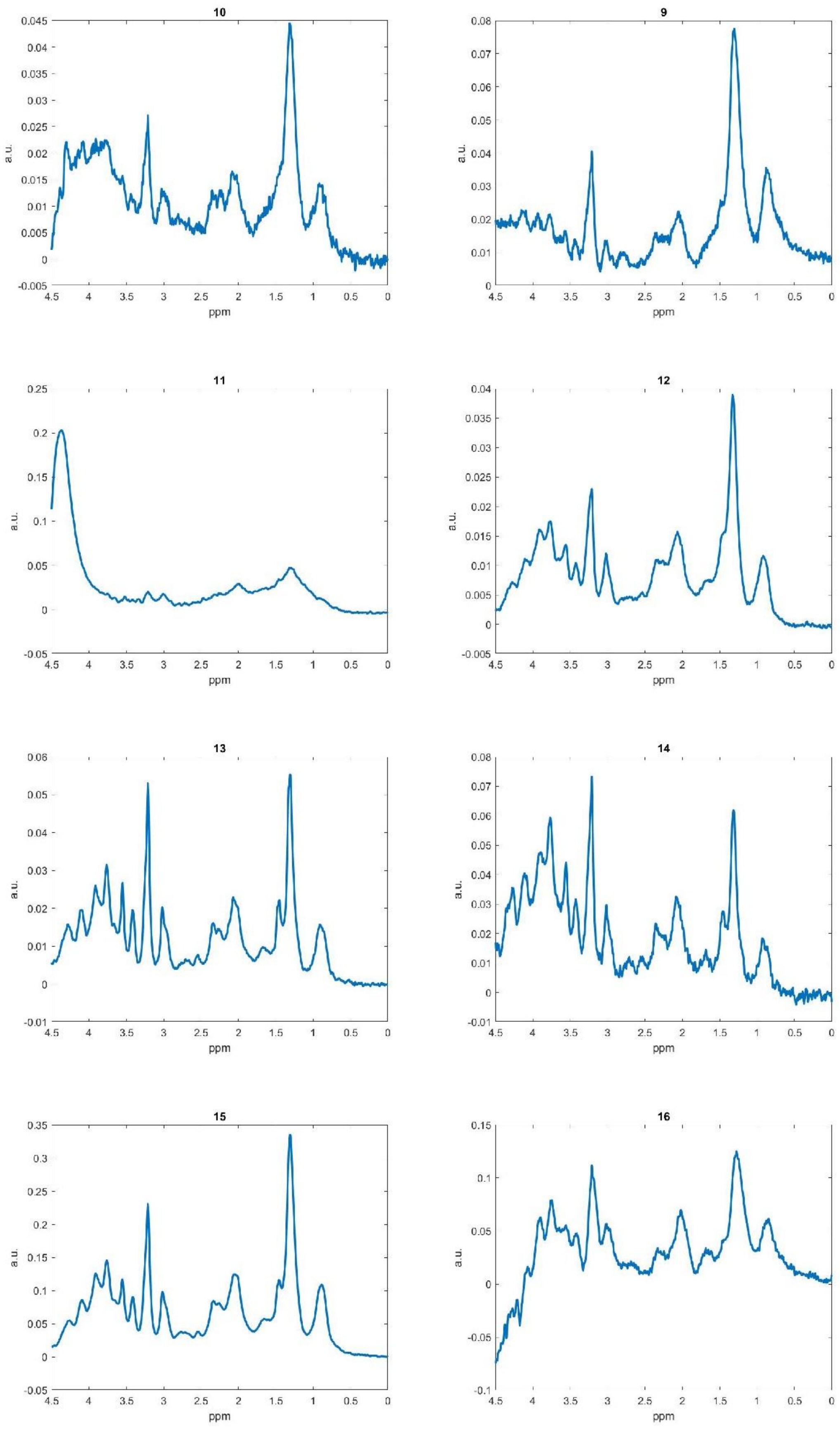
10
9
11
12
13
14
15
16
a.u.
ppm

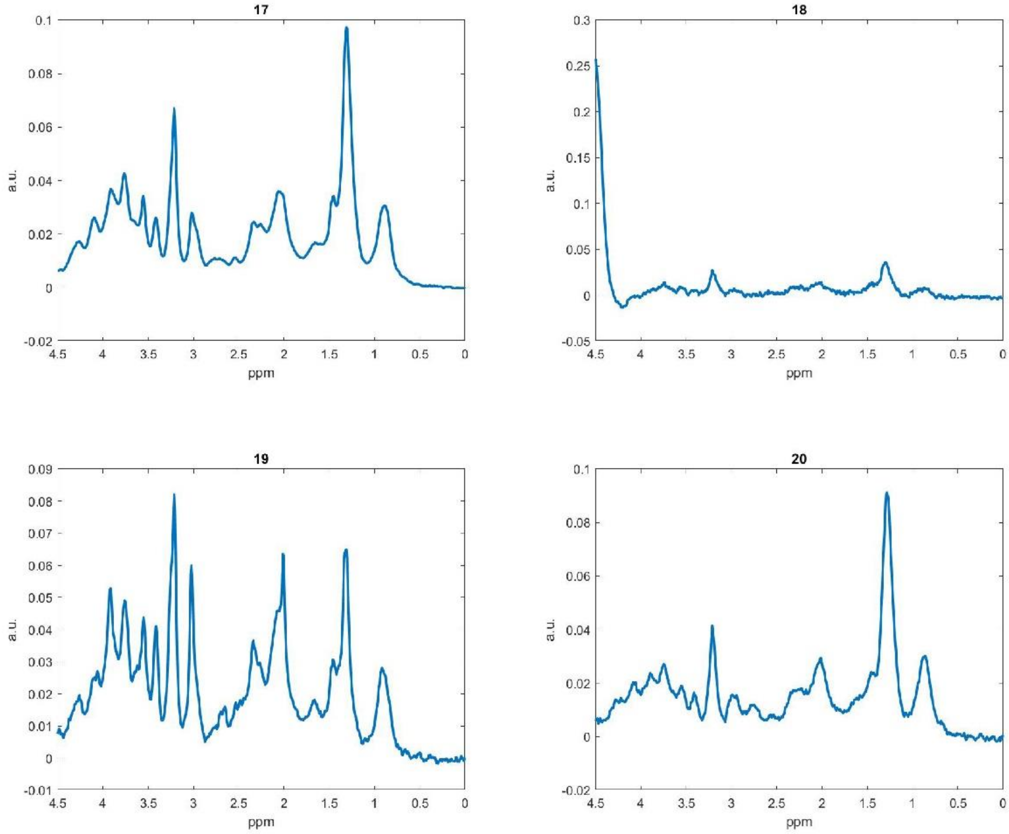

Figure 1: The 20 cNMF-extracted sources used in our experiments. Horizontal axis: frequency in parts per million (ppm: dimensionless units).

Ranking Position	*t*-Test method	Wrapper method
**1**	14	2
**2**	2	10
**3**	10	15
**4**	16	1
**5**	7	7
**6**	8	8
**7**	13	16
**8**	20	12
**9**	12	4
**10**	15	6
**11**	5	5
**12**	9	19
**13**	4	3
**14**	11	9
**15**	1	17
**16**	17	20
**17**	19	11
**18**	18	18
**19**	6	13
**20**	3	14

Table S4: cNMF sources ranking by t-test and wrapper methods.

RESULTS

The results graphically presented in Figures 1 and 2 of the main text are numerically summarized in *Tables S5, S6, S7* and *S8*.

Table S5 corresponds to Figure 1a in the main text and represents Radiomics results.

Table S6 corresponds to Figure 1b in the main text and represents Radiomics voting results.

Table S7 corresponds to Figure 2a in the main text and represents sources results.

Table S8 corresponds to Figure 2b in the main text and represents sources voting results.

Number of Features	Wrapper	*t*-test
1	0.6737	0.5684
2	0.7474	0.5895
3	0.6632	0.5579
4	0.7053	0.5684
5	0.6211	0.5579
6	0.6632	0.6000

7	0.6526	0.6105
8	0.6526	0.6000
9	0.6211	0.5895
10	0.6316	0.6211
11	0.6211	0.6211
12	0.6211	0.5895
13	0.6421	0.5684
14	0.6526	0.5474
15	0.6316	0.5684
16	0.6632	0.5368
17	0.6632	0.5368
18	0.6526	0.6105
19	0.6737	0.6316
20	0.6737	0.6105
21	0.6526	0.6105
22	0.6526	0.6105
23	0.6421	0.5474
24	0.6632	0.5684
25	0.6632	0.5368
26	0.6632	0.5263
27	0.6737	0.4842
28	0.6632	0.4737
29	0.6526	0.4632
30	0.6526	0.5158

Table S5: Radiomics results

Number of Features	Wrapper	*t*-test
1	0.7742	0.5484
2	0.7742	0.5484
3	0.7419	0.6452
4	0.7097	0.6129
5	0.6129	0.5806
6	0.6452	0.6774
7	0.7097	0.8065
8	0.7097	0.6452
9	0.6452	0.8387
10	0.6452	0.8710
11	0.6452	0.7419
12	0.6452	0.7419
13	0.6452	0.6452
14	0.6452	0.6774
15	0.6129	0.6774
16	0.6452	0.7097
17	0.6452	0.6774

18	0.6452	0.8065
19	0.6452	0.8387
20	0.6452	0.8065
21	0.6452	0.7742
22	0.6774	0.7419
23	0.6452	0.6129
24	0.6452	0.5806
25	0.6452	0.6452
26	0.6452	0.6452
27	0.6774	0.5161
28	0.6774	0.4839
29	0.6774	0.4839
30	0.6774	0.5484

Table S6: Radiomics voting results

Number of Features	Wrapper	*t*-test
1	0.5016	0.5686
2	0.5330	0.5627
3	0.5765	0.5960
4	0.5838	0.5772
5	0.5907	0.5907
6	0.6431	0.6431
7	0.6458	0.6448
8	0.8176	0.6154
9	0.8203	0.7586
10	0.8216	0.7820
11	0.8219	0.7929
12	0.8219	0.7902
13	0.8219	0.7886
14	0.8160	0.7916
15	0.8163	0.8150
16	0.8166	0.8193
17	0.8173	0.8199
18	0.8176	0.8206
19	0.8203	0.8160
20	0.8331	0.8160

Table S7: Sources results

Number of Features	Wrapper	*t*-test
1	0.4839	0.6452
2	0.4839	0.7097
3	0.5161	0.7419
4	0.5484	0.6452
5	0.5484	0.6774
6	0.6774	0.7097
7	0.7097	0.6452
8	0.9032	0.6452
9	0.9032	0.8710
10	0.9032	0.8710
11	0.9032	0.8710
12	0.9032	0.8710
13	0.9032	0.8710
14	0.9032	0.8710
15	0.9032	0.9032
16	0.9032	0.9032
17	0.9032	0.9032
18	0.9032	0.9032
19	0.9032	0.9032
20	0.9032	0.9032

Table S8: Sources voting results
